# Characterization of the efficacies of osimertinib and nazartinib against cells expressing clinically relevant epidermal growth factor receptor mutations

**DOI:** 10.18632/oncotarget.22297

**Published:** 2017-11-06

**Authors:** Keita Masuzawa, Hiroyuki Yasuda, Junko Hamamoto, Shigenari Nukaga, Toshiyuki Hirano, Ichiro Kawada, Katsuhiko Naoki, Kenzo Soejima, Tomoko Betsuyaku

**Affiliations:** ^1^ Division of Pulmonary Medicine, Department of Medicine, Keio University School of Medicine, Tokyo 160-8582, Japan; ^2^ Keio Cancer Center, Keio University School of Medicine, Tokyo 160-8582, Japan

**Keywords:** EGFR tyrosine kinase inhibitors, EGFR mutations, osimertinib, nazartinib, lung cancer

## Abstract

Third-generation epidermal growth factor receptor (EGFR) tyrosine kinase inhibitors (EGFR-TKIs) were developed to overcome *EGFR* T790M-mediated resistance to first- and second-generation EGFR-TKIs. Third-generation EGFR-TKIs, such as osimertinib and nazartinib, are effective for patients with the *EGFR* T790M mutation. However, there are no direct comparison data to guide the selection of a third-generation EGFR-TKI for patients with different *EGFR* mutations. We previously established an *in vitro* model to estimate the therapeutic windows of EGFR-TKIs by comparing their relative efficacies against cells expressing mutant or wild type EGFRs. The present study used this approach to characterize the efficacy of third-generation EGFR-TKIs and compare them with that of other EGFR-TKIs. Treatment efficacy was examined using human lung cancer-derived cell lines and Ba/F3 cells, which were transduced with clinically relevant mutant EGFRs. Interestingly, mutation-related differences in EGFR-TKI sensitivity were observed. For classic *EGFR* mutations (exon 19 deletion and L858R, with or without T790M), osimertinib showed lower IC50 values and wider therapeutic windows than nazartinib. For less common *EGFR* mutations (G719S or L861Q), afatinib showed the lowest IC50 values. For G719S+T790M or L861Q+T790M, the IC50 values of osimertinib and nazartinib were around 100 nM, which was 10- to 100-fold higher than those for classic+T790M mutations. On the contrary, osimertinib and nazartinib showed similar efficacies in cells expressing EGFR exon 20 insertions. The findings highlight the diverse mutation-related sensitivity pattern of EGFR-TKIs. These data may help in the selection of EGFR-TKIs for non-small cell lung cancer patients harboring *EGFR* mutations.

## INTRODUCTION

Lung cancer is the leading cause of cancer-related death worldwide [1]. A significant proportion of non-small cell lung cancers (NSCLCs) carry epidermal growth factor receptor (*EGFR*) gene mutations [2–6], which have been identified in approximately 10–30% of NSCLCs [7, 8]. Somatic mutation of the EGFR tyrosine kinase domain promotes the active conformation of this receptor, hence inducing its constitutive activation [9–11]. Activated EGFR transduces downstream signals, including the extracellular signal-regulated kinase (ERK)/mitogen-activated protein kinase (MAPK) pathway, and phosphatidylinositol-3-kinase/protein kinase B (AKT) [12–14]. The most common or “classic” mutations are in-frame deletions around the LREA motif in exon 19, which account for approximately 45% of *EGFR* mutations, and the exon 21 L858R point mutation, accounting for approximately 40% of *EGFR* mutations. Other less common *EGFR* mutations include G719X (3% of *EGFR* mutations), L861Q (2% of *EGFR* mutations) [12], and exon 20 insertion mutations (4–10% of *EGFR* mutations) [15–17].

EGFR tyrosine kinase inhibitors (EGFR-TKIs) have been developed to inhibit EGFR-mediated signaling. These molecules bind reversibly or irreversibly to the ATP binding pocket of EGFR, thus inhibiting activation. The exon 19 deletions, the L858R, the G719X, and the L861Q mutations result in sensitivity to the first-generation EGFR-TKIs, gefitinib and erlotinib. The response rates to gefitinib or erlotinib among NSCLC patients harboring the classic *EGFR* mutations are around 60–80% [7, 18]. Acquired resistance can occur after treatment with first- or second-generation EGFR-TKIs and the *EGFR* T790M mutation accounts for about 60% of this resistance [19, 20]. EGFR T790M is thought to induce resistance to these EGFR-TKIs by decreasing the affinity of EGFR-TKIs and increasing the affinity of ATP to tyrosine kinase domain ATP binding pocket of EGFR [20, 21]. Third-generation EGFR-TKIs irreversibly bind to the EGFR ATP binding pocket via a covalent interaction with the C797 residue, thereby blocking the increased affinity for ATP conferred by the EGFR T790M mutation. Some of third-generation EGFR-TKIs such as osimertinib [22]and nazartinib, which was formerly called EGF816, have demonstrated clinically significant efficacy and safety in NSCLC patients harboring *EGFR* T790M mutations, although the development of other third-generation candidates (rociletinib, olmutinib and naquotinib) has been halted [23, 24]. Osimertinib treatment produced a high objective response rate of approximately 60% for tumors with T790M mutations that showed resistance to first-generation EGFR-TKIs [22]. Nazartinib is undergoing clinical evaluation [25].

Unlike classic *EGFR* mutations, there is a paucity of data regarding the EGFR-TKI sensitivity of patients with lung cancers expressing less common EGFR mutations. For tumors expressing some of these mutations such as G719X, L861Q, and S768I, afatinib was effective [26]. However, the efficacy of third-generation EGFR-TKIs in patients with these mutations, in the presence or absence of the T790M mutation, is unclear.

On the other hand, most *EGFR* exon 20 insertion mutations confer resistance to first- and second-generation EGFR-TKIs [15, 27, 28]. One exception is *EGFR* A763_Y764insFQEA, which we previously reported as a first-generation EGFR-TKI-sensitizing mutation [29]. Our previous study reported the potential efficacy of osimertinib against tumors with exon 20 insertions associated with first- and second-generation EGFR-TKI resistance [30]. We created an *in vitro* model to determine the therapeutic windows for EGFR-TKIs, where the ratios of the 50% inhibitory concentrations (IC50) in Ba/F3 cells transduced with either mutated or wild type *EGFRs* were calculated [30]. We identified a therapeutic window of osimertinib for several *EGFR* exon 20 insertion mutations. The efficacy of nazartinib against cells expressing EGFR exon 20 insertion mutations has also been reported previously [31]. These authors revealed that nazartinib potently inhibited major subtypes of exon 20 insertion mutations, with EC50 values of 7, 11, and 190 nmol/L against D770_V771dupSVD, V769_D770insASV, and H773_V774insNPH, respectively. Furthermore, they showed an antitumor effect of nazartinib in a mouse patient-derived xenograft model expressing H773_V774insNPH. Therefore, osimertinib and nazartinib are expected to be potentially effective for tumors with exon 20 insertion mutations.

There are no direct comparison data relating to the efficacies of EGFR-TKIs for tumors expressing different EGFR mutations, including the less common mutations. The present study aimed to characterize the efficacy of third-generation EGFR-TKIs and to compare them with that of other EGFR-TKIs using *in vitro* modeling. This analysis of the efficacy of EGFR-TKIs provides preclinical information to inform the proper selection of EGFR-TKIs for the treatment of patients with cancers harboring *EGFR* mutations.

## RESULTS

### Therapeutic windows for osimertinib and nazartinib in Ba/F3 cells expressing classic EGFR mutations, in the presence or absence of T790M

**Figure 1:**
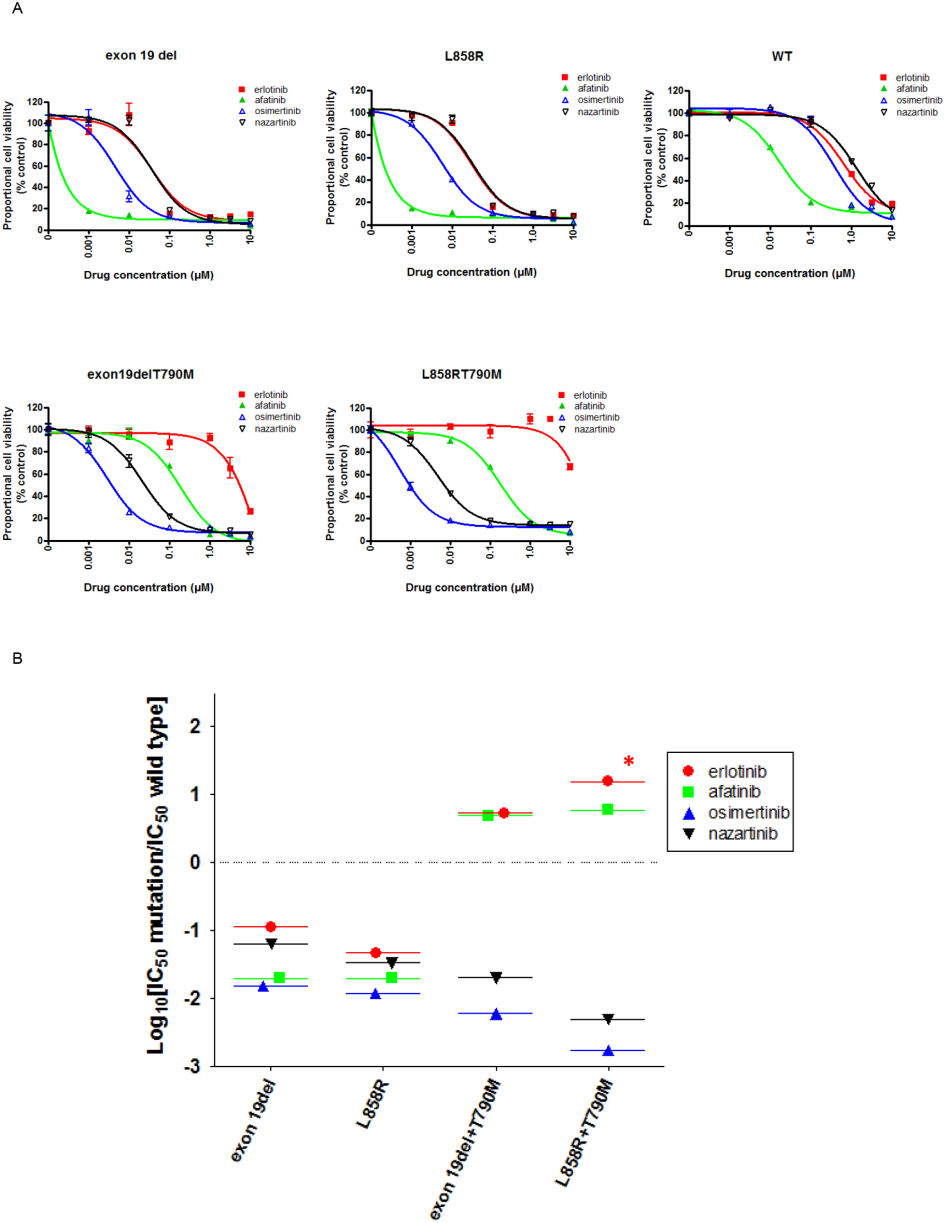
The sensitivity of Ba/F3 cells expressing wild type (WT) or mutant EGFR to EGFR-TKIs **(A)** MTS assays were conducted in Ba/F3 cells expressing the indicated EGFR genotypes. Data points represent the mean ± standard deviation. **(B)** Selectivity index (SI) values were calculated for cells expressing the indicated EGFR mutations versus wild type.^*^; SI index >1.

We generated stable EGFR-expressing Ba/F3 cell lines in order to directly compare the sensitivities of multiple *EGFR* mutations to EGFR-TKIs using MTS assays (Figure 1A). The viabilities of Ba/F3 cells harboring exon 19del or L858R were dramatically reduced by afatinib. Erlotinib also effectively inhibited the proliferation of these cells, although it was less potent than afatinib. The potency of nazartinib was comparable to that of erlotinib, while osimertinib was more potent than either erlotinib or nazartinib. Although low concentrations of erlotinib did not inhibit the proliferation of Ba/F3 cells harboring *EGFR* T790M mutations (*EGFR* exon 19del + T790M or L858R + T790M), afatinib, osimertinib, and nazartinib effectively reduced the viability of these cells.

In the clinic, skin rashes and diarrhea are common or life-threatening side effects of EGFR-TKIs. These side effects are most likely caused by the inhibition of wild type EGFR expressed in epithelial cells within the lungs, gastrointestinal tract, and skin. Hence, the clinical utility of EGFR-TKIs is hampered by their lack of selectivity for mutated versus wild type EGFR; this results in a narrow therapeutic window, with a high level of toxicity at the clinical doses required to effectively inhibit mutated EGFR. We therefore evaluated the effects of EGFR-TKIs on wild type EGFR using the MTS assay. Wild type EGFR-expressing Ba/F3 cells were efficiently inhibited by afatinib, while low concentrations of nazartinib did not inhibit their viability. The IC50 value for afatinib was 30 nM, as compared to 1031 nM for nazartinib, indicating that afatinib had more potent effects on wild type EGFR. The IC50 values for each EGFR-TKI examined in this study are summarized in Table 1.

**Table 1:** IC_50_ values (nM) of Ba/F3 cells expressing EGFR mutations

Mutations	erlotinib	afatinib	osimertinib	nazartinib
exon 19del	73	0.6	7.9	66
L858R	30	0.6	6.2	35
G719S	101	1.5	158	91.2
L861Q	410	3.6	35.8	116
A763_Y764insFQEA	152	<0.1	24	78
Y764_V765insHH	2948	140	143	387
A767_V769dupASV	3073	77	134	257
D770_N771insNPG	2342	36	28	84
exon19del+T790M	3429	146	3.1	52
L858R+T790M	>10000	179	0.9	5.1
G719S+T790M	>10000	34	97	77
L861Q+T790M	>10000	163	73	101
exon19del+T790M+C797S	5153	390	655	2146
L858R+T790M+C797S	8360	826	1024	3834
WT	638	30	516	1031

We used *in vitro* modeling to calculate the selectivity index (SI): the ratio of IC50 values in Ba/F3 cells transduced with mutant or wild type *EGFRs* (Figure 1B). In this model, a low SI value indicates a highly mutant *EGFR*-selective EGFR-TKI. For Ba/F3 cells harboring exon 19del or L858R, osimertinib showed the lowest SI values of the EGFR-TKIs included in this study; this indicated a wide therapeutic window for osimertinib in patients with these mutations. Nazartinib showed slightly higher SI values than osimertinib in cells expressing classic EGFR mutations. For Ba/F3 cells harboring *EGFR* T790M, osimertinib and nazartinib showed striking mutation specificity. Their SI values of ≤ −2 indicated IC50 values for cells expressing the T790M mutation that were > 100-fold lower than those for cells expressing wild type EGFR. On the other hand, erlotinib and afatinib did not demonstrate mutation specificity for *EGFR* T790M mutations. These SI values were > 0, indicating IC50 values for cells expressing EGFR T790M were higher than those for cells expressing wild type EGFR.

### Effect of EGFR-TKIs on EGFR downstream signals and apoptosis in cells Ba/F3 expressing classic EGFR mutations in the presence or absence of the T790M mutation

To examine EGFR signaling pathways, we performed immunoblotting (Supplementary Figure 1A). In Ba/F3 cells harboring exon 19del or L858R, afatinib dramatically inhibited the phosphorylation of EGFR, AKT, and ERK. Erlotinib, osimertinib, and nazartinib inhibited the phosphorylation of EGFR, AKT, and ERK to a similar extent. In Ba/F3 cells harboring the *EGFR* T790M mutation (exon 19del + T790M or L858R + T790M), erlotinib did not inhibit the phosphorylation of EGFR, AKT, and ERK. However, afatinib, osimertinib, and nazartinib did effectively inhibit the phosphorylation of these molecules. These data indicated that the sensitivity of Ba/F3 cells to EGFR-TKIs was reflected by the inhibition of the EGFR and its downstream signaling pathways.

Next, to biologically confirm the therapeutic windows for third-generation EGFR-TKIs in cells expressing the T790M mutation, we analyzed apoptosis in Ba/F3 cells expressing wild type EGFR or EGFR exon 19del + T790M. We stained these cells with annexin V-APC and propidium iodide after 48-h exposures to the indicated EGFR-TKIs (0.1 μM). The proportions of annexin V-positive and/or propidium iodide-positive cells were examined by flow cytometry (Supplementary Figure 1B). As expected, afatinib induced apoptosis in Ba/F3 cells harboring either wild type EGFR or EGFR exon 19del + T790M, indicating a narrow therapeutic window for afatinib in the presence of EGFR exon 19del + T790M. The proportion of annexin V-positive wild type EGFR-expressing cells was 91.0%, as compared with 32.8% of EGFR exon 19del + T790M-expressing cells. In contrast, osimertinib and nazartinib produced only minor effects on apoptosis in Ba/F3 cells harboring wild type *EGFR*, and were more effective in cells harboring *EGFR* exon 19del + T790M. The proportions of annexin V-positive wild type EGFR-expressing cells were 27.9% and 25.5%, respectively, in the presence of osimertinib and nazartinib, while the equivalent values for cells expressing EGFR exon 19del + T790M were 72.5% and 62.8%, respectively. These data indicated a wide therapeutic window for osimertinib and nazartinib in the treatment of tumors expressing the EGFR T790M mutation.

### Investigation of the sensitivity/resistance profiles of EGFR-TKIs in lung cancer cell lines

**Figure 2:**
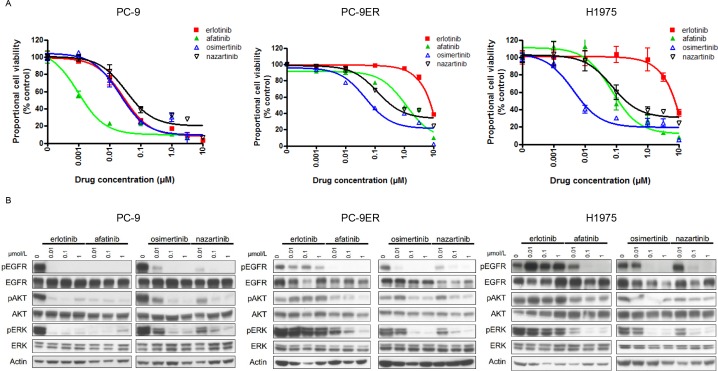
The sensitivities of lung cancer cell lines to EGFR-TKIs **(A)** MTS assays of PC-9, PC9-ER, and H1975 cells exposed to the indicated EGFR-TKIs. Data points represent the mean ± standard deviation. **(B)** PC-9, PC9-ER, and H1975 cells were treated with the indicated concentrations of EGFR-TKIs for 4 h prior to immunoblotting for the phosphorylated (p) and non-phosphorylated forms of EGFR, AKT, and ERK. Actin was used as a loading control.

To explore the relevance of the sensitivity/resistance profiles derived from stably transduced Ba/F3 cells, we also performed MTS assays in human lung cancer-derived cell lines in the presence and absence of EGFR-TKIs (Figure 2A). The lung cancer cell lines included in this study were PC-9 (*EGFR* exon 19del), PC-9ER (*EGFR* exon 19del + T790M), and H1975 (*EGFR* L858R + T790M). For PC-9 cells, afatinib produced the most dramatic inhibitory effect, with an IC50 value of 1.3 nM. Although less potent than afatinib, erlotinib also effectively inhibited the proliferation of PC-9 cells, with an IC50 value of 28 nM. The potencies of osimertinib and nazartinib were comparable to that of erlotinib in this cell line, with IC50 values of 23 nM and 36 nM, respectively. Low concentrations of erlotinib did not inhibit the proliferation of cell lines harboring the *EGFR* T790M mutation (PC-9ER and H1975). However, afatinib and the third-generation EGFR-TKIs, osimertinib and nazartinib, effectively inhibited the proliferation of these lung cancer cells. The calculated IC50 values of afatinib, osimertinib, and nazartinib for PC-9ER cells were 677 nM, 166 nM, and 276 nM, respectively. The calculated IC50 values of afatinib, osimertinib, and nazartinib for H1975 cells were 80 nM, 4.6 nM, and 52 nM, respectively. These IC50 values are summarized in Supplementary Table 1. These data indicated that the sensitivity/resistance profile data produced in stably transfected Ba/F3 cells were comparable to those generated using human lung cancer cell lines.

Furthermore, immunoblotting was performed to determine whether the sensitivity of lung cancer cells to EGFR-TKIs was mediated through inhibition of EGFR signaling (Figure 2B). Consistent with the results of the MTS assay, afatinib caused the most potent inhibition of the phosphorylation of EGFR and its downstream proteins, AKT and ERK, in PC-9 cells. Although they were less potent than afatinib, erlotinib, osimertinib, and nazartinib also effectively inhibited the phosphorylation of EGFR, AKT, and ERK in PC-9 cells. Our study of lung cancer cells harboring the *EGFR* T790M mutation showed that all of the tested EGFR-TKIs, except for erlotinib, effectively inhibited the phosphorylation of the EGFR, AKT, and ERK. In summary, these data indicated that the sensitivity/resistance profiles observed in Ba/F3 cells were also present in human lung cancer cells.

### Therapeutic windows of nazartinib and osimertinib in Ba/F3 cells expressing EGFR exon 20 insertion mutations

**Figure 3:**
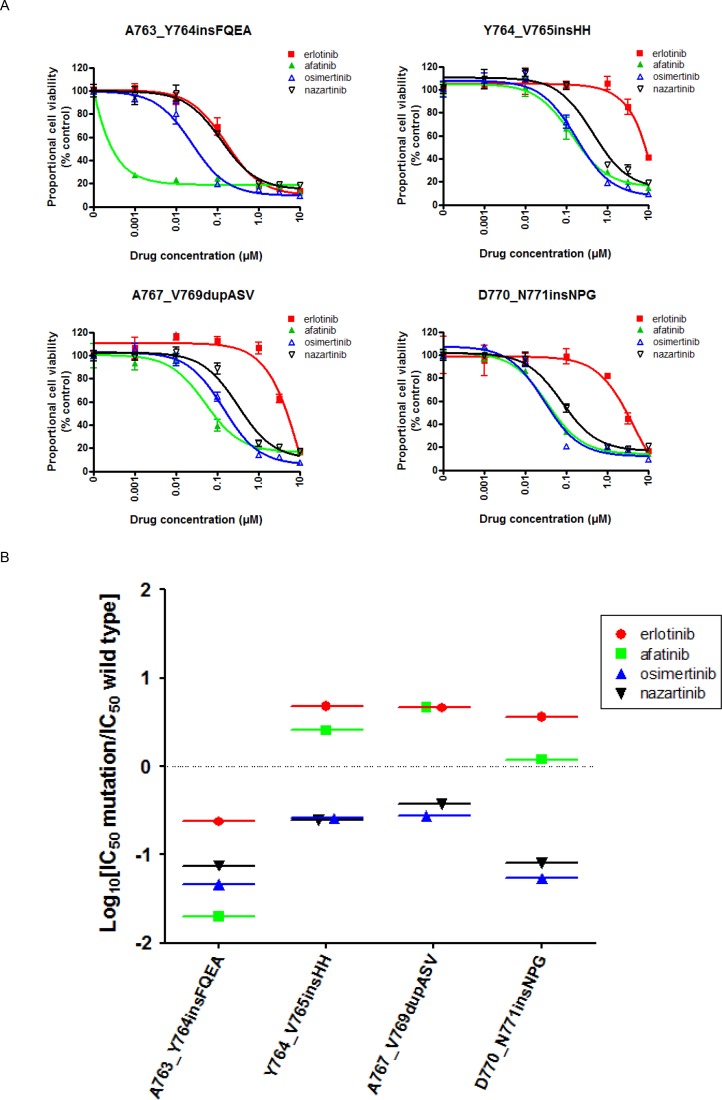
The EGFR-TKI sensitivity of Ba/F3 cells harboring the indicated *EGFR* genotypes **(A)** MTS assays were conducted in Ba/F3 cells harboring the indicated *EGFR* exon 20 insertion mutations. Data points represent the mean ± standard deviation. **(B)** The selectivity index (SI) values are shown for the indicated EGFR-TKIs in Ba/F3 cells expressing the indicated EGFR exon 20 insertion mutations.

We investigated the effects of EGFR-TKIs on the viability of Ba/F3 cells transduced with four representative *EGFR* exon 20 insertion mutations (A763_Y764insFQEA, Y764_V765insHH, A767_V769dupASV, and D770_N771insNPG) using MTS assays (Figure 3A). All of the tested EGFR-TKIs inhibited the growth of Ba/F3 cells harboring *EGFR* A763_Y764insFQEA. A similar response pattern was observed in a human lung cancer-derived cell line (BID007) harboring *EGFR* A763_Y764insFQEA (Supplementary Figure 2). Afatinib, osimertinib, and nazartinib showed comparable efficacies against cells expressing other first- and second-generation EGFR-TKI-resistant EGFR exon 20 insertion mutations, Y764_V765insHH, A767_V769dupASV, and D770_N771insNPG. The calculated IC50 values are summarized in Table 1. We applied the aforementioned *in vitro* model to estimate the therapeutic windows for EGFR-TKIs in cells expressing EGFR exon 20 insertion mutations (Figure 3B). In Ba/F3 cells harboring *EGFR* A763_Y764insFQEA, all EGFR-TKIs showed comparable SI values of around −1. In contrast, erlotinib and afatinib had SI values > 0 in cells expressing EGFR exon 20 insertion mutations associated with resistance to first- and second-generation EGFR-TKIs. In these cell lines, osimertinib and nazartinib had SI values of around −1, indicating IC50 values of about 10-fold lower than those observed in cells expressing wild type EGFR. These data indicated that both osimertinib and nazartinib could have therapeutic windows and act effectively against cells expressing these exon 20 insertion mutations.

### Effects of EGFR-TKIs on EGFR downstream signals and apoptosis in cells expressing EGFR exon 20 insertion mutations

We performed immunoblotting to investigate whether the sensitivity of Ba/F3 cells harboring *EGFR* exon 20 insertion mutations was associated with inhibition of the EGFR signaling pathway (Supplementary Figure 3A). As expected, afatinib dramatically inhibited the phosphorylation of EGFR, AKT, and ERK in cells expressing EGFR A763_Y764insFQEA. Osimertinib and nazartinib produced similar inhibitions of EGFR, AKT, and ERK phosphorylation. These inhibition patterns were also observed in BID007 cells (Supplementary Figure 3B). In cells expressing other EGFR exon 20 insertion mutations associated with resistance to first- and second-generation EGFR-TKIs (Y764_V765insHH, A767_V769dupASV, and D770_N771insNPG), afatinib, osimertinib, and nazartinib produced similar inhibition patterns.

We performed assays to determine the level of apoptosis in Ba/F3 cells harboring Y764_V765insHH and A767_V769dupASV, following exposure to 0.1 μM afatinib, osimertinib, or nazartinib for 48 h. The proportions of annexin V- and/or propidium iodide-positive cells were determined by flow cytometry (Supplementary Figure 4). In cells expressing Y764_V765insHH, the proportions of annexin V-positive cells following exposure to afatinib, osimertinib, or nazartinib were 64.2%, 72.9%, or 51.3%, respectively. The corresponding proportions in cells expressing A767_V769dupASV were 88.5%, 73.3%, or 48.9%, respectively. These data indicated that afatinib, osimertinib, and nazartinib induced apoptosis in cells expressing different EGFR exon 20 insertion mutations.

### Therapeutic windows of osimertinib and nazartinib in Ba/F3 cells expressing EGFR G719S, L861Q, and/or T790M

**Figure 4:**
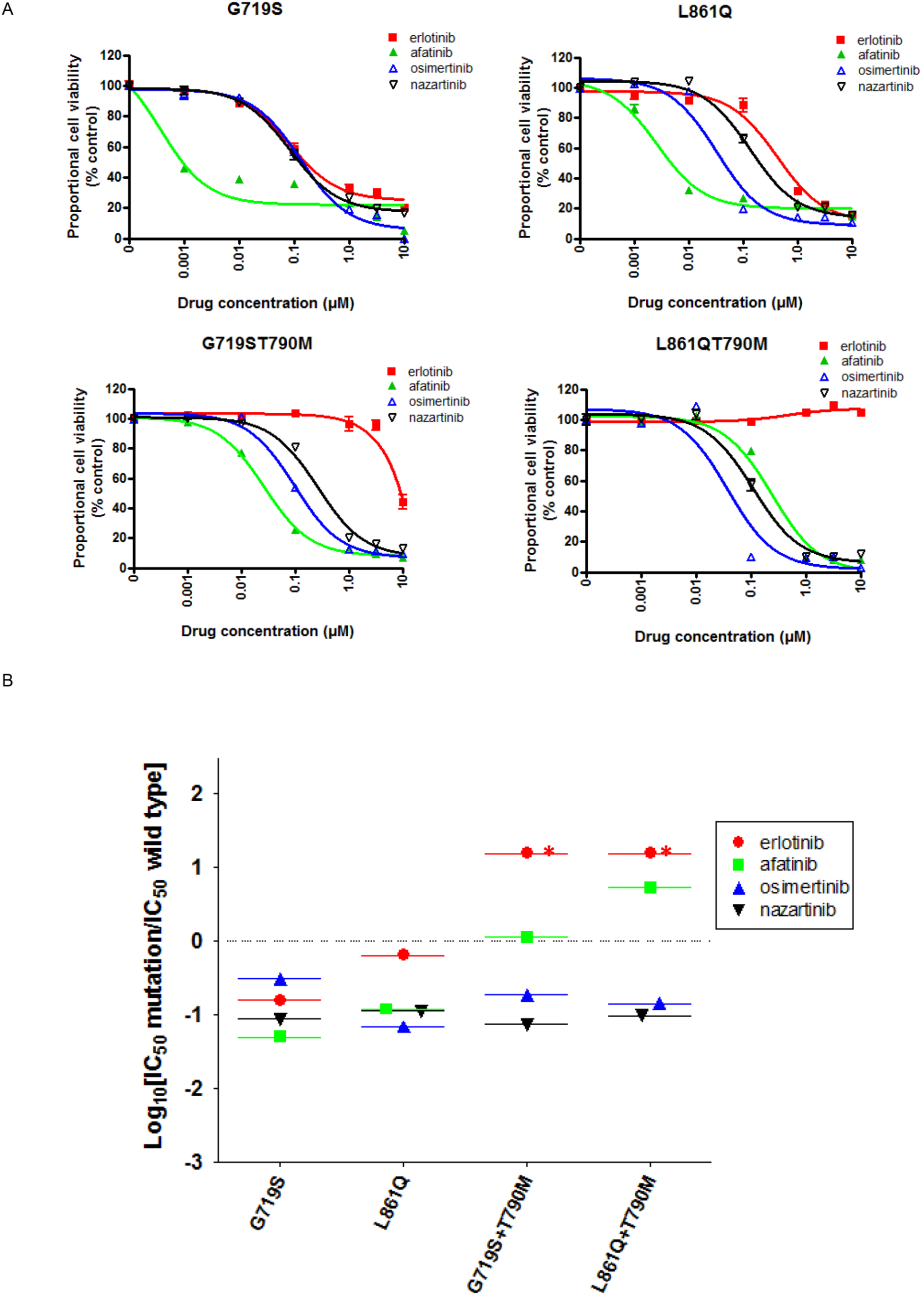
The EGFR-TKI sensitivity of Ba/F3 cells harboring the indicated *EGFR* genotypes **(A)** MTS assays were conducted in Ba/F3 cells harboring the indicated *EGFR* uncommon mutations, G719S and L861Q in the presence or absence of T790M. Data points represent the mean ± standard deviation. **(B)** The selectivity index (SI) values are shown for the indicated EGFR-TKIs in Ba/F3 cells expressing the indicated EGFR uncommon mutations. ^*^; SI index >1.

We used MTS assays to investigate the effects of EGFR-TKIs on the viability of Ba/F3 cells transduced with the less common *EGFR* mutations, G719S and L861Q, in the presence or absence of T790M (Figure 4A). The resultant IC50 values are summarized in Table 1. The viability of Ba/F3 cells harboring G719S and L861Q mutations was dramatically reduced by afatinib. For these mutations, the SI values of all EGFR-TKIs were < 0, and afatinib showed the lowest SI values in cells expressing G719S (Figure 4B). In the presence of T790M, the sensitivities to erlotinib and afatinib reduced. For G719S+T790M or L861Q+T790M, the IC50 values of osimertinib and nazartinib were around 100 nM, 10- to 100-fold higher than those observed in cells expressing classic+T790M mutations (Table 1). Moreover, the SI values of osimertinib and nazartinib for these mutations were around −1 (Figure 4B), higher than those observed in cells expressing classic mutations. These data indicate the possibility that third-generation EGFR-TKIs would have lower efficacies in tumors expressing the less common EGFR mutations, as compared with those expressing the classic EGFR mutations, regardless of T790M mutation.

### Effects of EGFR-TKIs on EGFR downstream signals and apoptosis in cells expressing EGFR G719S, L861Q, and/or T790M

We performed immunoblotting to investigate the effect of EGFR-TKIs on the EGFR signaling pathway (Supplementary Figure 5A). Afatinib dramatically inhibited the phosphorylation of EGFR, AKT, and ERK, particularly in cells expressing EGFR G719S+T790M. Osimertinib and nazartinib produced similar inhibitions of EGFR, AKT, and ERK phosphorylation in cells expressing G719S+T790M and L861Q+T790M. The level of apoptosis in Ba/F3 cells harboring these mutations were consistent with the IC50 values for these EGFR-TKIs (Supplementary Figure 5B).

### The efficacy of osimertinib in NSCLC patients harboring G719X in the presence or absence of T790M

**Figure 5:**
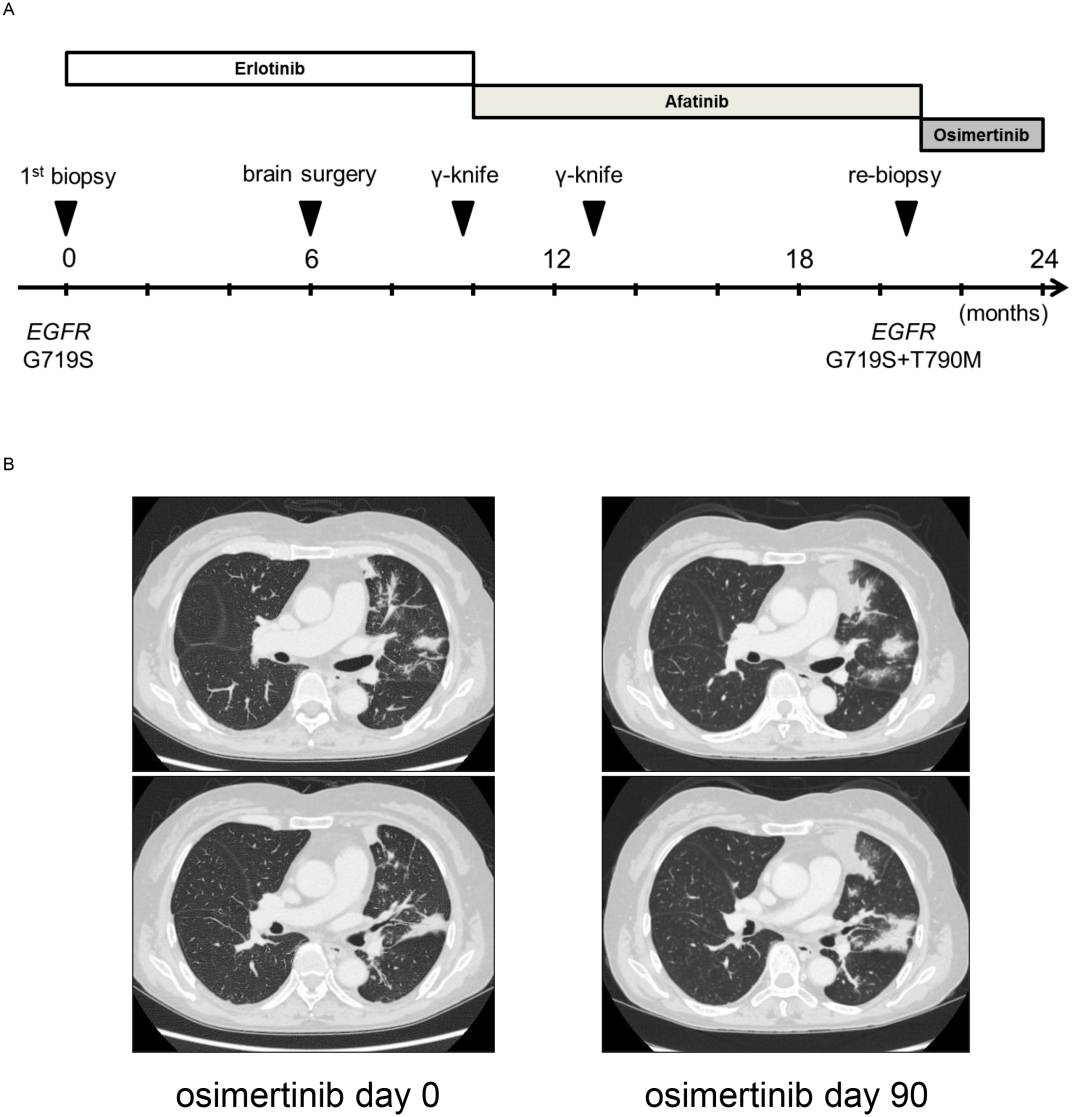
**(A)** Clinical course after diagnosis of NSCLC. **(B)** Clinical response to osimertinib in a patient with NSCLC harboring *EGFR* G719S+T790M. Chest computed tomography images of the patient were captured before and after osimertinib treatment.

To seek the clinical relevance of the obtained data about the G719X mutation, we reviewed clinical data. In the AURA trial, a phase I/II clinical trial (NCT01802632), a total of seven patients with the G719X mutation received at least one dose of osimertinib. *EGFR* T790M was detected in three of these patients. Of the 7 patients, 1 (14%) had a confirmed partial response, 3 (43%) had stable disease, and 3 (43%) had progressive disease. In addition, we identified an interesting case in our institution. A 63-year-old woman was diagnosed with stage IV adenocarcinoma of the lung via biopsy using bronchoscopy. This tumor expressed the EGFR G719S mutation. The timeline of her anticancer treatment is shown in Figure 5A. Treatment with erlotinib maintained stable pulmonary lesions, but did not control her brain metastases; she therefore underwent γ-knife and brain surgery. After 10 months, the left upper lobe tumor had expanded and she received afatinib as a second-line therapy. After 11 months, the left upper lobe lesion enlargement led to a second computed tomography-guided biopsy. Molecular analysis found *EGFR* G719S and T790M mutations. The patient was then treated with osimertinib (80 mg/day). However, after 3 months, she met the definition of progressive disease (+ 127%) because the left upper lobe tumor had expanded (Figure 5B) and left adrenal gland involvement was detected (not shown). These findings may reflect the limited efficacy of third-generation EGFR-TKIs for tumors expressing G719X, in the presence or absence of T790M.

### EGFR-TKI resistance in cells expressing the EGFR C797S mutation

To date, several mechanisms underlying acquired resistance to third-generation EGFR-TKIs have been reported in preclinical models and clinical specimens; these include the tertiary *EGFR* C797S mutation [32–35] that prevents the covalent binding of EGFR-TKIs, *EGFR* L718Q mutation [36, 37], MET amplification [31, 32], HER2 amplification [32], mutated *EGFR* allele amplification [38], or wild type *EGFR* allele amplification [39]. Cys797 is the amino acid that is covalently modified by third-generation EGFR-TKIs and its substitution by Ser797 abolishes this interaction. We investigated the effects of EGFR-TKIs on the viability of Ba/F3 cells harboring delL747_P753insS + T790M + C797S (exon 19del + T790M + C797S) or L858R + T790M + C797S using MTS assays (Supplementary Figure 6A). None of the tested EGFR-TKIs inhibited the proliferation of these cells at low concentrations. The IC50 values of EGFR-TKIs for *EGFR* C797S-positive cell lines are summarized in Table 1. Furthermore, the SI values of the tested EGFR-TKIs were > 0 (Supplementary Figure 6B). In summary, these data confirmed that Ba/F3 cells expressing the *EGFR* C797S mutation were resistant to these EGFR-TKIs.

## DISCUSSION

The present study compared the effects of erlotinib, afatinib, osimertinib, and nazartinib on cells expressing a range of EGFR mutations; these included classic mutations (exon 19 deletions and L858R), the T790M mutation, less common mutations (G719S, L861Q and exon 20 insertion mutations), and the C797S mutation. The therapeutic window for these EGFR-TKIs is an important determinant of their potential toxicity in humans. To quantify this, we used our previously reported *in vitro* model to determine the ratio of their IC50 values in Ba/F3 cells transduced with mutant or wild type *EGFRs* [30]. In general, the SI values obtained in this study were tightly matched to previously published data [30], indicating the robustness of this model.

In addition, the findings of our studies in stably transduced Ba/F3 cell lines and in human lung cancer-derived cell lines were consistent. All the tested EGFR-TKIs demonstrated wide therapeutic windows in cells expressing the classic EGFR mutations, exon 19 deletions and L858R. Interestingly, osimertinib had a wider therapeutic window than nazartinib. On the contrary, in cells expressing the EGFR T790M mutation, osimertinib and nazartinib showed markedly wider therapeutic windows than either erlotinib or afatinib. These data indicate that osimertinib and nazartinib could provide effective and safe EGFR-TKIs for patients with *EGFR* T790M-positive tumors. This is consistent with human clinical trials, in which osimertinib and nazartinib have shown promising safety and efficacy [22].

Unlike classic *EGFR* mutations, there is a paucity of data regarding the EGFR-TKI sensitivity of patients with lung cancers expressing less common EGFR mutations. Preclinical data have indicated that *EGFR* exon 18 mutations (exon 18 deletions, E709K, and G719A) and others (S768I and L861Q) exhibited higher sensitivity to afatinib than to erlotinib or osimertinib [40, 41]. In the present study, afatinib had a wider therapeutic window for G719S than for L861Q. A combined post-hoc analysis of LUX-Lung 2, LUX-Lung 3, and LUX-Lung 6 found that the response rate to afatinib was higher for G719X (77.8%) than for L861Q (56.3%) [26]. Furthermore, we identified limited efficacy of third-generation EGFR-TKIs for these mutations, irrespective of the T790M mutation. Interestingly, osimertinib was not effective for the patient with NSCLC harboring the G719S+T790M mutations. In the AURA trial, 7 patients with G719X-positive tumors received at least one dose of osimertinib. The objective response rate was 14% and the disease control rate was 57% [22]. In summary, the efficacy of third-generation EGFR-TKIs is likely to be lower in patients with the *EGFR* G719X mutation than in those with *EGFR* classic mutations, both in the presence and absence of the T790M mutation. Osimertinib and nazartinib showed similar potencies and greater mutation specificity than afatinib in cells expressing exon 20 insertion mutations, which are associated with resistance to first- and second-generation EGFR-TKIs. Dose-adjusted clinical trials will be required to examine the efficacy of osimertinib or nazartinib for patients with NSCLCs harboring these types of exon 20 insertion mutations.

Structurally, the *EGFR* T790M mutation induces resistance to first- and second-generation EGFR-TKIs by altering the gatekeeper residue in the ATP-site and increasing the affinity for ATP [21]. The *EGFR* C797S mutation impairs the covalent binding between the cysteine residue at position 797 of EGFR and third-generation EGFR-TKIs [33]. In general, the data obtained in this study was reasonable from the structural view point.

The present study was conducted *in vitro*. To develop improved strategies for using EGFR-TKIs, further *in vivo* and human clinical trials will be required because EGFR-TKIs may have different pharmacokinetic and pharmacodynamic properties that will influence their *in vivo* concentrations.

In summary, the present study provides fundamental osimertinib and nazartinib sensitivity/resistance data in cells expressing a range of EGFR mutations, including those that occur relatively infrequently. The findings highlight the diverse mutation-related sensitivity to EGFR-TKIs. These data may help in the selection of EGFR-TKIs for the treatment of NSCLC patients.

## MATERIALS AND METHODS

### Cell lines

Four human NSCLC cell lines were used: PC-9 [*EGFR* exon 19 deletion (delE746-A750)]; PC-9ER [*EGFR* exon 19 deletion (delE746-A750) + T790M]; BID007 [*EGFR* exon 20 insertion (A763_Y764insFQEA)]; and H1975 [*EGFR* L858R + T790M]. PC9 and BID007 cells were a kind gift from Dr. Susumu Kobayashi (Beth Israel Deaconess Medical Center, Boston, MA, USA). H1975 cells were purchased from the American Type Culture Collection (Manassas, VA, USA). PC-9ER cells became resistant to erlotinib after chronic exposure to this molecule and acquisition of the *EGFR* T790M mutation. Cell authentication for H1975 was performed in June 2015.

### Reagents

Erlotinib and afatinib were purchased from LC Laboratories (Woburn, MA, USA). Osimertinib was purchased from Selleck Chemicals (Houston, TX, USA). Nazartinib (#B5889) was purchased from Apexbio. Antibodies recognizing total EGFR (#2232), total AKT (#9272), phospho-AKT (S473; D9E; #4060), total p44/42 MAPK (#9102S), and phospho-p44/42 MAPK (T202/204; #9101S) were purchased from Cell Signaling Technology (Beverly, MA, USA). The phospho-EGFR (Y1068) antibody (44788G) was purchased from Invitrogen/Life Technologies (Carlsbad, CA, USA), and the actin antibody was purchased from Sigma-Aldrich (St. Louis, MO, USA).

### Ba/F3 stable cell lines

Ba/F3 cells stably expressing either wild type or mutated EGFR were created as previously described [29]. Ba/F3 cells harboring *EGFR* mutations were cultured in RPMI-1640 growth medium, supplemented with 10% fetal bovine serum, at 37°C in a humidified 5% CO_2_ incubator. Ba/F3 cells expressing wild type EGFR were cultured in RPMI-1640 growth medium, supplemented with 10% fetal bovine serum, at 37°C in a humidified 5% CO_2_ incubator with EGF (10 ng/mL). The *EGFR* mutations examined in this study included: delL747_P753insS (exon 19del); L858R; delL747_P753insS + T790M (exon 19del + T790M); L858R + T790M; A763_Y764insFQEA; Y764_V765insHH; A767_V769dupASV; D770_N771insNPG; G719S; L861Q; G719S + T790M; L861Q + T790M; delL747_P753insS + T790M + C797S (exon 19del + T790M + C797S); and L858R + T790M + C797S.

### Cell proliferation assay

The MTS (3-(4,5-dimethylthiazol-2-yl)-5-(3-carboxymethoxyphenyl)-2-(4-sulfophenyl)-2H-tetrazolium) assay was performed as previously described [29]. PC-9, PC-9ER, H1975, and BID007 cells were seeded in 96-well plates. Twenty-four hours after seeding, the appropriate medium with or without EGFR-TKI was added to each well. Control cells were treated with the same concentration of the vehicle, dimethyl sulfoxide (DMSO). Seventy-two hours after treatment, absorbance was measured. For Ba/F3 cells, the cells were seeded with or without EGFR-TKI. Seventy-two hours after seeding, absorbance was measured. All experiments were performed at least three times.

### Immunoblotting analysis

Cells were treated with EGFR-TKI at concentrations of 0.01–1 μmol/L for 4 h. Cells were lysed in Cell Lysis Buffer (Cell Signaling Technology). Equal amounts of protein were loaded per lane on sodium dodecyl sulfate-polyacrylamide gels. Separated proteins were transferred to polyvinylidene fluoride membranes. The membranes were incubated overnight with primary antibodies at 4°C and then incubated with secondary antibodies for 1 h. For the detection of proteins, the membranes were incubated with agitation in LumiGLO reagent and peroxide (Cell Signaling Technology), and then exposed to X-ray film.

### Apoptosis assay

Ba/F3 cells harboring wild type *EGFR*, exon 19del + T790M, Y764_V765insHH, A767_V769dupASV, G719S + T790M, or L861Q + T790M were seeded in 6-well plates. The cells were treated with EGFR-TKIs (0.1 μM) for 48 h. Control cells were treated with the same concentration of the vehicle, DMSO. We analyzed the apoptotic status of cells using the Annexin V Apoptosis Detection Kit APC (eBioscience, San Diego, CA, USA), according to the manufacturer's protocol. The proportion of apoptotic cells was evaluated by flow cytometric analysis, using the Gallios flow cytometer system (Beckman Coulter, Brea, CA, USA).

### Statistical analysis

Statistical analysis was performed using the GraphPad Prism software, version 4.0 (GraphPad Software, La Jolla, CA, USA). The IC_50_ values were calculated using GraphPad Prism software.

### Human data

This study included NSCLC patient data. The study was reviewed and approved by the Institutional Review Board of Keio University School of Medicine (Tokyo, Japan). Tumor samples were obtained with written informed consent. *EGFR* mutation analyses were performed on genomic DNA extracted from tumor samples by using the PNA-LNA PCR clamp method.

## SUPPLEMENTARY MATERIALS FIGURES AND TABLE


