# Supplementary material for: Characterization of the efficacies of osimertinib and nazartinib against cells expressing clinically relevant epidermal growth factor receptor mutations
Source: Oncotarget. 2017 Nov 6;8(62):105479–91. doi: 10.18632/oncotarget.22297 (PMC5739653; doi:10.18632/oncotarget.22297)
Supplement: Supplementary file 1 [file oncotarget-08-105479-s001.pdf]

Characterization of the efficacies of osimertinib and nazartinib against cells expressing clinically relevant epidermal growth factor receptor mutations

SUPPLEMENTARY MATERIALS

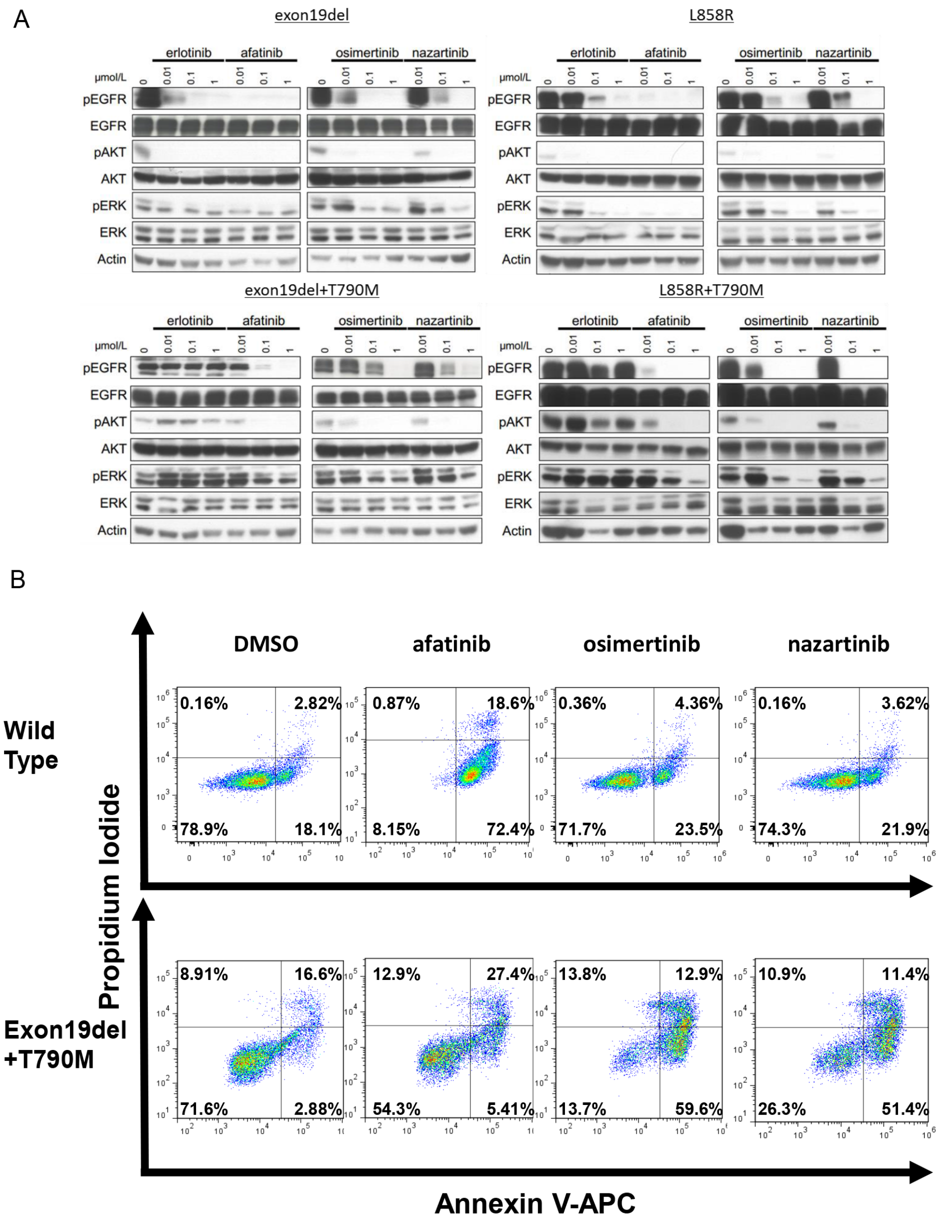

Supplementary Figure 1: The effects of EGFR-TKIs on EGFR downstream signals and apoptosis in Ba/F3 cells expressing the indicated EGFR genotypes. (A) Ba/F3 cells expressing the indicated EGFR genotypes were treated with the indicated concentrations of EGFR-TKIs for 4 h prior to immunoblotting for the phosphorylated (p) and non-phosphorylated forms of EGFR, AKT, and ERK. Actin was used as a loading control. **(B)** Apoptosis assays were conducted using flow cytometry in Ba/F3 cells harboring the indicated EGFR genotypes and treated with EGFR-TKIs for 48 h prior to staining with propidium iodide and annexin V-APC. The numbers indicate the percentages of cells in the annexin V- and/or propidium iodide-positive quadrants.

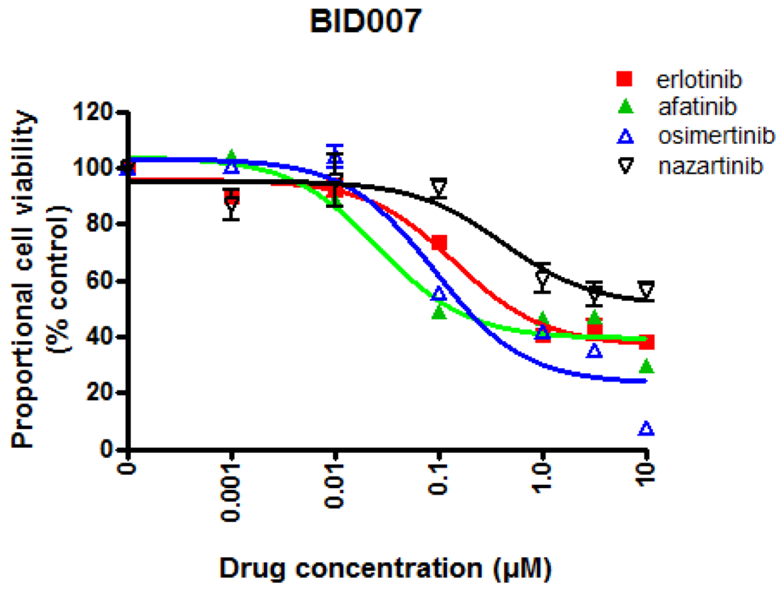

Supplementary Figure 2: MTS assay for BID007 (EGFR A763_Y764insFQEA) cells. Error bars indicate standard deviation.

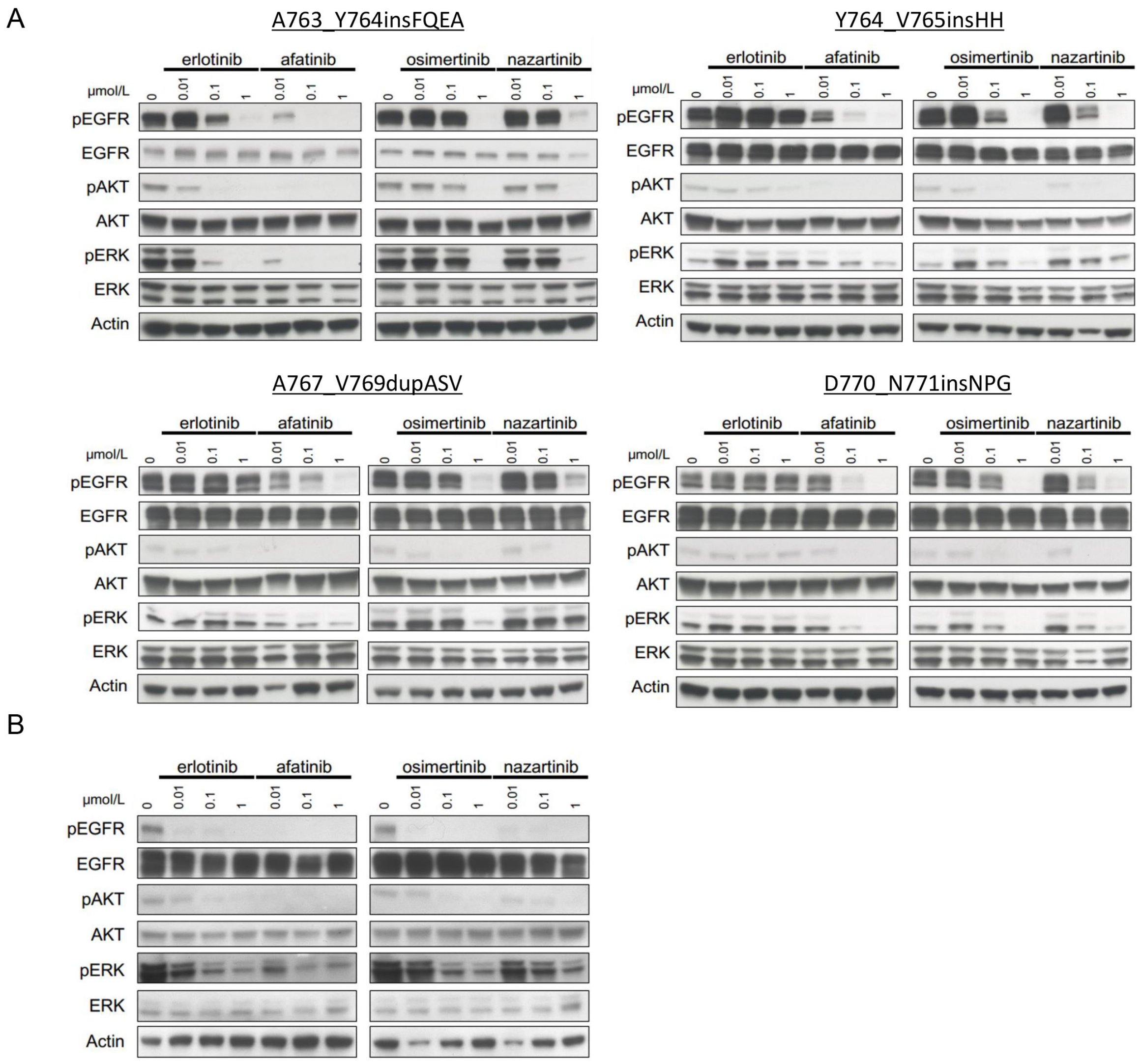

Supplementary Figure 3: (A) Results of immunoblotting for Ba/F3 cells is shown. The cells were treated with the indicated concentrations of EGFR-TKIs for 4 h. Erlotinib, afatinib, osimertinib, and nazartinib were used as EGFR-TKIs. pEGFR, pAKT, and pERK indicate the phosphorylated form of EGFR, AKT, and ERK, respectively. Actin was used as a loading control. **(B)** Results of immunoblotting for BID007 cell is shown. The cell was treated with the indicated concentrations of EGFR-TKIs for 4 h. Erlotinib, afatinib, osimertinib, and nazartinib were used as EGFR-TKIs. pEGFR, pAKT, and pERK indicate the phosphorylated form of EGFR, AKT, and ERK, respectively. Actin was used as a loading control.

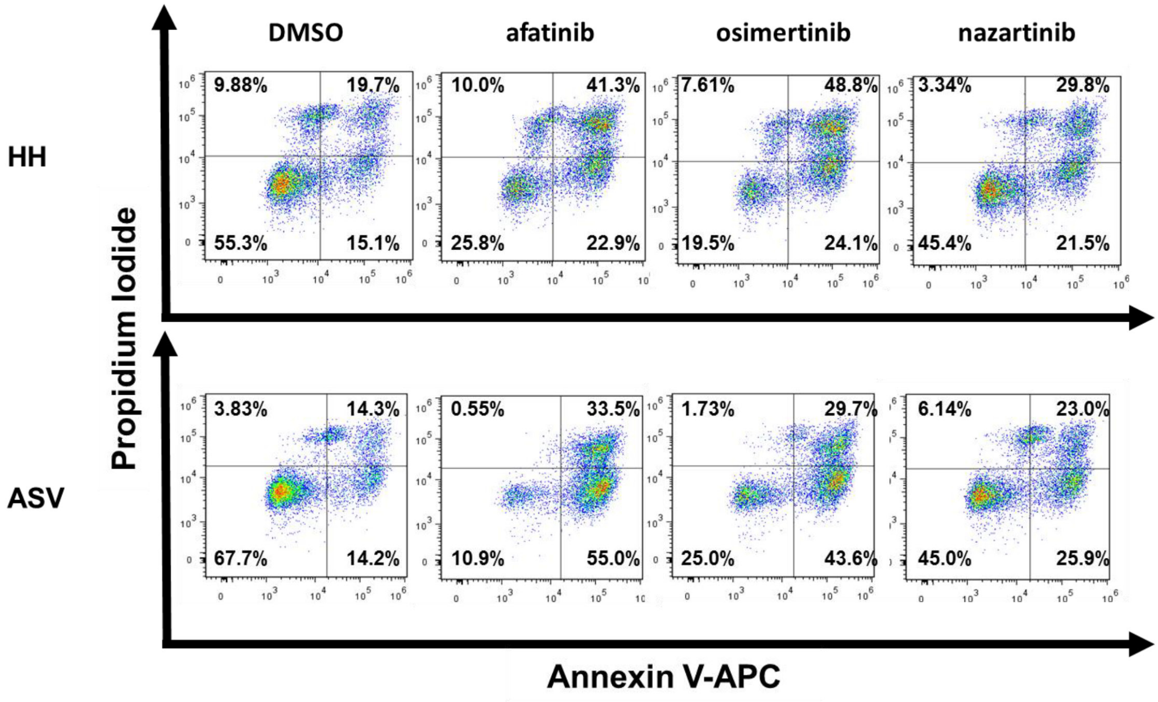

Supplementary Figure 4: Apoptosis assays were conducted using flow cytometry in Ba/F3 cells harboring the Y764_V765insHH (HH) and A767_V769dupASV (ASV) following treatment with EGFR-TKIs for 48 h, and staining with propidium iodide and annexin V-APC. The numbers indicate the percentages of cells in the annexin V- and/or propidium iodide-positive quadrants.

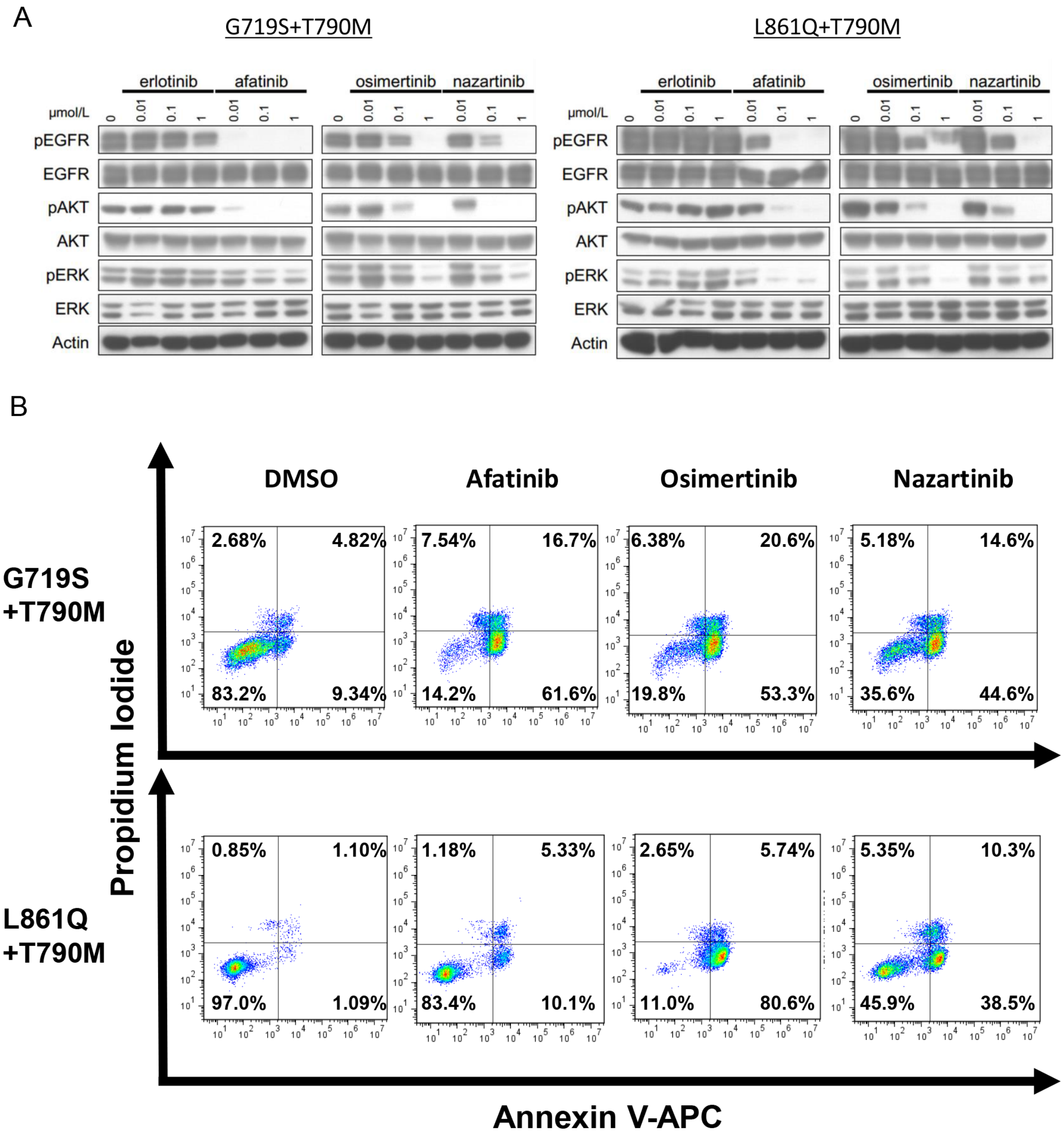

Supplementary Figure 5: The effects of EGFR-TKIs on EGFR downstream signals and apoptosis in cells expressing G719S and L861Q mutation with T790M mutation. **(A)** Immunoblotting was conducted in Ba/F3 cells expressing EGFR G719S and L861Q mutation with T790M mutation. The cells were treated with the indicated concentrations of EGFR-TKIs for 4 h. pEGFR, pAKT, and pERK indicate the phosphorylated forms of EGFR, AKT, and ERK, respectively. Actin was used as a loading control. **(B)** Apoptosis assays were conducted using flow cytometry in Ba/F3 cells harboring the indicated *EGFR* genotypes following treatment with EGFR-TKIs for 48 h, and staining with propidium iodide and annexin V-APC. The numbers indicate the percentages of cells in the annexin V- and/or propidium iodide-positive quadrants.

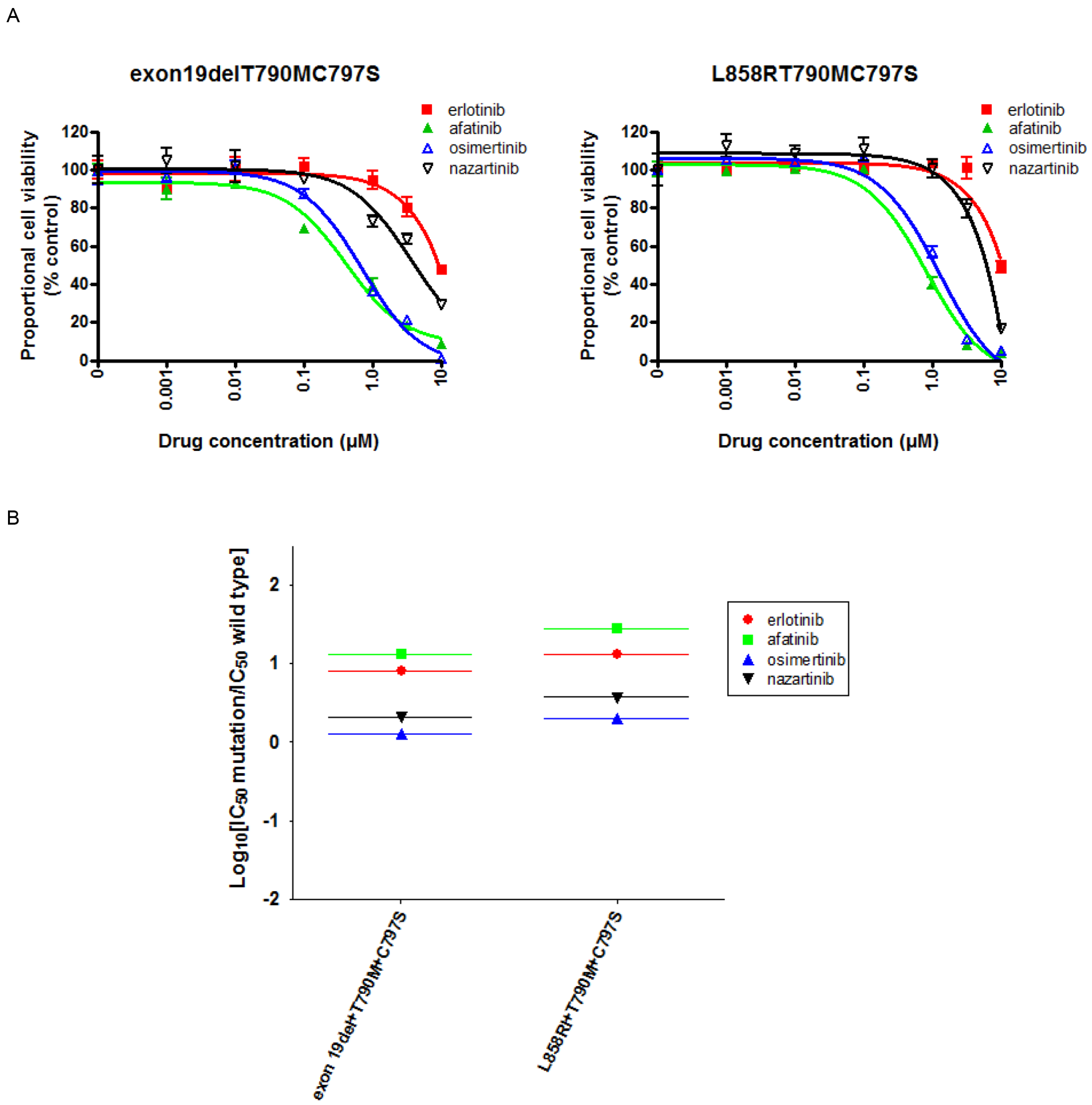

Supplementary Figure 6: The sensitivity of Ba/F3 cells harboring *EGFR* C797S mutations to EGFR-TKIs. (A) MTS assays were conducted in Ba/F3 cells harboring the *EGFR* exon 19 deletion + T790M + C797S or L858R + T790M + C797S. Data points represent the mean ± standard deviation. **(B)** The selectivity index (SI) values are shown for the indicated EGFR-TKIs in Ba/F3 cells expressing the indicated *EGFR* genotype.

Supplementary Table 1: IC_{50} values (nM) of lung cancer cell lines

	erlotinib	afatinib	osimertinib	nazartinib
PC-9 (exon 19del)	28	1.3	23	36
PC-9ER (exon 19del+T790M)	7963	677	166	276
H1975 (L858R+T790M)	3342	80	4.6	52
BID007 (A763_Y764insFQEA)	134	26	73	488
